# Rapid review of evaluation of interventions to improve participation in cancer screening services

**DOI:** 10.1177/0969141316664757

**Published:** 2016-10-17

**Authors:** Stephen W Duffy, Jonathan P Myles, Roberta Maroni, Abeera Mohammad

**Affiliations:** Centre for Cancer Prevention, Wolfson Institute of Preventive Medicine, Barts and The London School of Medicine and Dentistry, Queen Mary University of London, London, UK

**Keywords:** Breast cancer, cancer screening, cervical cancer, colorectal cancer, ethnicity, intervention, participation, reminder, review, socioeconomic status, uptake

## Abstract

**Objective:**

Screening participation is spread differently across populations, according to factors such as ethnicity or socioeconomic status. We here review the current evidence on effects of interventions to improve cancer screening participation, focussing in particular on effects in underserved populations.

**Methods:**

We selected studies to review based on their characteristics: focussing on population screening programmes, showing a quantitative estimate of the effect of the intervention, and published since 1990. To determine eligibility for our purposes, we first reviewed titles, then abstracts, and finally the full paper. We started with a narrow search and expanded this until the search yielded eligible papers on title review which were less than 1% of the total. We classified the eligible studies by intervention type and by the cancer for which they screened, while looking to identify effects in any inequality dimension.

**Results:**

The 68 papers included in our review reported on 71 intervention studies. Of the interventions, 58 had significant positive effects on increasing participation, with increase rates of the order of 2%–20% (in absolute terms).

**Conclusions:**

Across different countries and health systems, a number of interventions were found more consistently to improve participation in cancer screening, including in underserved populations: pre-screening reminders, general practitioner endorsement, more personalized reminders for non-participants, and more acceptable screening tests in bowel and cervical screening.

## Introduction

To achieve their desired public health impact, population cancer screening services require high levels of participation. While it is agreed that decisions to participate in cancer screening should be free from undue pressure, and should be well-informed, it is also frequently observed that there are considerable inequalities in participation in cancer screening.^1–3^ In the United Kingdom (UK), screening participation rates are lower in areas of deprivation and among certain ethnic groups.^3–5^ There is a wide range of potential interventions to improve access to cancer screening services and, therefore, increase participation. Given the perceived need to address health inequalities,^6^ one tactic would be to improve uptake of public health measures such as screening programmes in currently underserved populations. We review the evidence on effects of interventions to improve screening participation, with particular reference to effects on inequalities. To inform policy and practice, such a review should identify those measures most and least likely to be effective, including any findings with respect to inequalities, or effects of interventions in deprived or otherwise underserved populations.

## Methods

We specified in advance that the studies of relevance to this review had to report on interventions aimed at participation in cancer screening services (not randomized trials of screening). The focus of interest was population screening programmes (as opposed to surveillance of specific genetic or other high-risk groups). To be eligible, a study had to report a quantitative estimate of the effect of the intervention on participation rates. We only included studies published since 1990. Studies which assessed the effect of personal invitation against no invitation were not included, as the NHS Cancer Screening Programmes in the UK would always send personal invitations in any case.

The commissioners of this research required results in a relatively short time and resources were not available for the traditional systematic review. Therefore, rather than the customary search strategy which begins as comprehensively as possible and frequently identifies tens of thousands of populations, we started with a narrow search and expanded successively (by adding ‘OR’ terms) until the number of new publications eligible on title review comprised less than 1% of the total. The major assumption here was that if successive expansions of the search yield diminishing numbers of potentially eligible publications, and if the most recent expansion yields a relatively small addition to the pool, stopping the expansion at this point is unlikely to lead to a major loss of information. The successive searches and their outcomes are shown in Table 1. As this strategy is less comprehensive than the standard systematic review procedure, as a further safeguard, we also specified that the publications yielded by the final search had to include four recent studies which were clearly relevant.^7–10^ The other restriction was that only peer-reviewed results obtainable in PubMed searches were considered. Searches took place in late September 2015.

**Table 1.** Results of successively broadening the search terms until newly identified papers potentially eligible on title review was less than 1% of the total papers found by the search.

PubMed Search	Number of publications	Number of publications selected on title review	New publications potentially eligible after title review	Percentage of new publications potentially eligible
‘Cancer’ AND ‘Screening’ AND (‘Participation’ OR ‘Attendance’) AND (‘Interventions’ OR ‘Involvement’) AND ‘Invitation’	51	28	28	56%
‘Cancer’ AND ‘Screening’ AND (‘Participation’ OR ‘Attendance’) AND (‘Interventions’ OR ‘Intervention’ OR ‘Involvement’) AND ‘Invitation’	89	50	22	25%
‘Cancer’ AND ‘Screening’ AND (‘Participation’ OR ‘Attendance’ OR ‘Appointment’) AND ‘Invitation’	343	93	43	13%
‘Cancer’ AND ‘Screening’ AND (‘Participation’ OR ‘Attendance’ OR ‘Appointment’) AND (‘Invitation’ OR ‘Involvement’)	724	97	4	0.6%

Papers passing title review underwent abstract review. Those remaining eligible after abstract review underwent full paper review. In addition to original papers, our searches identified five reviews.^11–15^ From these, and from the reference lists of the papers eligible after full paper review, we identified further potential papers which, in turn, were subject to abstract and, if eligible, full paper review. Finally, colleagues identified evaluations of eight interventions published in three papers since the searches took place.^16–18^

**Figure 1.**
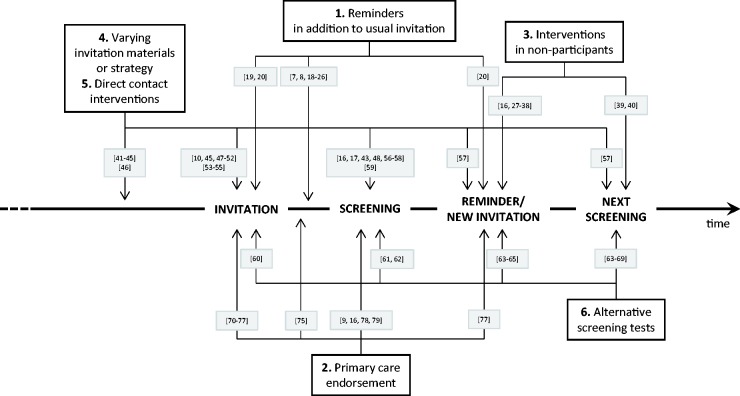
Schematic diagram showing the categories of intervention by time point on the screening pathway at which the intervention takes place, with references to the relevant studies in parentheses.

Papers were reviewed for the effect of the intervention by the cancer for which they were screening and by intervention type (reminders in addition to usual invitation; primary care endorsement; additional interventions in non-participants; enhanced invitation materials or varying invitation strategy; direct contact; varying the screening test). Interventions can occur at various stages of the process, from advance notice communications sent prior to first invitation to screening, to the offer of a different screening test at the screening episode some years following non-participation in a previous episode (see Figure 1). We deal with the arbitrary nature of the classification below (see Discussion section). In addition, interventions were studied to identify effects by different socioeconomic or ethnic grouping, if any, and to identify any other inequality dimension in the work.

In the description of results of interventions below, percentage increases in participation refer to absolute increases; for example, a 5% increase would refer to a difference between 20% and 25% (an absolute increase of 5%) rather than 20% and 21% (a 5% relative increase).

## Results

After abstract review, 62 papers were deemed eligible. On full paper review, two were excluded, leaving 60. From reference lists and reviews, after full paper review, further five papers were added. As noted above, colleagues identified another three papers published since the searches, bringing the total to 68 papers included, although one reports on four separate trials and is therefore included four times in the tables of results.^16^

### Reminders in addition to usual invitation

Results are shown in Table 2. Eleven papers were identified, two in colorectal, six in breast, two in cervical and one in stomach cancer screening.^7,8,18–26^ Ten of the 11 reported an increase in participation with reminders (3/3 text reminder studies; 4/5 telephone reminder studies; 2/2 postal reminder studies; 1/1 telephone + postal reminder study). The absolute magnitude of the effects was an increase in participation of the order of between 3 and 10 percentage points. Most studies were in urban areas, including substantial underserved and low socioeconomic status populations. All seven of the studies explicitly reporting results in such underserved populations found an increase in participation with the intervention.^7,8,18,20–23^

**Table 2.** Studies of reminders additional to usual invitation.

Publication and intervention	Region	Cancer	Design	Results	Inequality dimension
Baker et al.^21^ Postal and telephone reminders.	Chicago, USA	Colorectal	Randomized trial. 225 subjects randomized to usual care (opportunistic reminders), 225 to a series of postal and telephone reminders.	Significantly greater participation in intervention group (82% vs. 37%).	Majority of subjects were Spanish speaking and were uninsured.
Shankleman et al.^22^ Telephone reminders.	London, UK	Colorectal	Cluster-randomized trial. Nine general practices randomized to: (1) usual care (2) telephone reminder with health promotion information (3) face-to-face health promotion information.	Significant increase in participation with telephone intervention. Participation rates were: (1) 33.8% (2) 45.9% (3) 41.2%.	Study took place in inner north-east London, containing areas of deprivation and high ethnic diversity.
Kerrison et al.^7^ Text-message reminders.	London, UK	Breast	Single-blind randomized control trial of 2240 women receiving first breast screening invitation randomly assigned in a 1:1 ratio (normal invite vs. normal invite plus text reminder).	Sending women a text-message reminder significantly increased attendance (64% vs. 59%).	Odds ratio in favour if intervention highest in most deprived group. No formal test for heterogeneity.
Allgood et al.^18^ Postal reminders.	North-west England	Breast	Randomized trial of usual invitation (11,445 women) vs. usual invitation + postal reminder one week prior to appointment (11,383 women).	Significantly greater participation in reminder group (75% vs. 72%).	Study incorporated areas of considerable deprivation.
Offman et al.^8^ Telephone reminders.	Newham, London, UK	Breast	Observational study of planned intervention of 10,928 women invited for breast screening telephoned to: confirm receipt of the invitation letter, remind invitees of their upcoming appointment, and provide further information.	Reminder calls substantially improved breast cancer screening uptake (67% vs. an expected 57%).	Study took place in an inner city area with high ethnic diversity and considerable deprivation.
Arcas et al.^23^ Text-message reminders.	Barcelona, Spain	Breast	233 women received usual invitation (control group), 470 received an additional text-message reminder (intervention group).	Control group uptake 72%, intervention 78%.	Effect significant in those with low educational status.
Taplin et al.^24^ Telephone reminders and motivational telephone calls.	Seattle, USA	Breast	590 randomized to reminder postcard, 585 to reminder telephone call, and 590 to motivational telephone call addressing barriers, tailored to demographic features of the invitees.	Participation was 35% in the postcard group, 52% in the telephone reminder group, and 50% in the motivational call group.	Relatively affluent Caucasian population.
Vidal et al.^25^ Text-message reminders.	Catalonia, Spain	Breast	Non-randomized study. 3719 sent text message in addition to invitation, compared to 9067 receiving invitation only.	75% response with reminder, 65% without.	Effective in areas where postal service less reliable.
Acera et al.^19^ Four invitation strategies, including reminders.	Barcelona, Spain	Cervical	Cluster-randomized trial in 3225 women of: (1) no action (2) personalized invitation letter (3) personalized invitation letter + information leaflet (4) personalized invitation letter + leaflet + telephone reminder.	Coverage: (1) 8% (2) 51% (3) 59% (4) 52%.	Relatively low income and educational status of population.
Eaker et al.^20^ Telephone and postal reminders.	Sweden	Cervical	12,240 women randomized to: (1) modified invitation vs. standard invitation (2) reminder letter vs. no reminder letter (3) telephone contact of non-attenders vs. no telephone contact.	Significant 9% increase in attendance with reminder letter. 31% increase with telephone reminder. Results: (1) 27% vs. 26% (2) 16% vs. 6% (3) 41% vs. 10%.	Effect stronger in less deprived groups.
Lee et al.^26^ Postal reminders.	Goyang, South Korea	Stomach	1262 men randomized to: (1) no intervention (2) telephone counselling (3) telephone counselling followed by mailed reminder (4) mailed reminder followed by telephone counselling.	Any intervention significantly improved attendance in never-screened men. Attendance among never screened by group: (1) 10% (2) 14% (3) 36% (4) 15%.	Not clear.

### Primary care endorsement

Results are shown in Table 3. Twelve studies were identified, six in colorectal, four in breast, and two in cervical cancer screening.^9,16,70–79^ Ten of the 12 reported positive results. One of the studies which did not find increased participation was in the context of a flexible sigmoidoscopy project in the UK before flexible sigmoidoscopy was included in the national programme, and rather than a letter with general practitioner (GP) endorsement, the intervention was the offer of a consultation with the GP to discuss the screening.^70^ Typical increases in participation of the order of 2%–3% were observed, but some studies found increases of 10%–20%. All four studies explicitly reporting the effect in underserved populations found an increase in participation with the intervention.^16,72,75,76^ One study of a multilingual intervention aimed at ethnic minorities in Cardiff found an increase in participation in people of south Asian origin, higher in Gujarati and Urdu speakers than in Bengali speakers.^72^

**Table 3.** Primary care endorsement studies.

Publication and intervention	Region	Cancer	Design	Results	Inequality dimension
Wardle et al.^16^ General practice endorsement as a banner across invitation letter.	England	Colorectal	Cluster randomized trial. 134,011 invitees randomized to be sent usual invitation, 131,423 letter with GP endorsement banner.	Increased participation with GP endorsement (58% vs. 57%). Non-significant trend of increasing effect in more deprived populations.	Formally designed to investigate significant difference in effect among deprivation categories.
Hewitson et al.^9^ Primary care endorsement letter and a patient leaflet.	South of England, UK	Colorectal	Randomized controlled 2 × 2 factorial trial of 1288 patients randomized to either a GP's endorsement letter and/or an enhanced information leaflet with their FOBT kit.	Including both an endorsement letter from each patient's GP and a more explicit procedural leaflet increased participation. 61% vs. 49% usual care.	Conducted in an area of medium to high socioeconomic status.
Gray et al.^70^ Letter offering opportunity to discuss flexible sigmoidoscopy screening with GP.	Scotland, UK	Colorectal	Patients aged between 50.5 and 60.5 randomly allocated to one of two groups. The first group was sent an invitation to have screening sigmoidoscopy along with an explanatory leaflet. The second group was sent the same invitation and leaflet but with an added option to discuss the test in the first instance with their GP.	The overall uptake rate was 24%. Significantly fewer people in the second group replied to the initial invitation.	Urban setting. Otherwise, none reported.
Barthe et al.^71^ Invitation signed by GP.	France	Colorectal	Cluster randomized by GP (57 GP's). 1895 patients sent invitation signed by GP, 1527 usual invitation.	Uptake: 15% in each group.	Urban setting. Otherwise, none reported.
Zajac et al.^78^ Letter with primary care endorsement.	South Australia	Colorectal	Randomized study. 1200 offered FIT without GP endorsement, 600 with GP endorsement mentioned in accompanying letter (GP2), and 600 with invitation explicitly from own practice (GP3).	Significant increase in participation in those with explicit invitation from own general practice. At first round, results were no endorsement, 33%; GP1, 39%; GP2, 42%.	None reported.
Cole et al.^79^ Letter with primary care endorsement.	South Australia	Colorectal	Similar to Zajac, above. 600 offered FOBT without GP endorsement, 600 with GP endorsement mentioned in accompanying letter (GP2), and 600 with invitation explicitly from own practice (GP3).	Significantly higher participation and re-participation over subsequent screening rounds in both GP-endorsed groups. At first round: no endorsement, 32%; GP2, 38%; GP3, 41%.	None reported.
Bell et al.^72^ Multiple intervention including GP endorsement.	Cardiff, UK	Breast	369 women in three general practices with a low uptake in the previous round of breast screening and a high proportion of ethnic minority women on their lists received GP endorsement letter, multilingual leaflet, offer of transport to the screening centre and language support.	50.7% attendance compared with 32.5% in previous round.	Uptake highest in Gujarati and Urdu speakers, lowest in Bengali and Somali speakers.
Giorgi et al.^73^ GP involvement and economic incentives to GP's.	Four cities in Italy	Breast	Varying among cities, comparison of GP invitation letters with screening centre letters. 20,087 women and 145 GP's.	Significantly higher participation with GP letters. 56% vs. 52% in Florence. 76% vs. 74% in Modena.	None reported.
Dorsch et al.^74^ GP invitation from GP lists.	South Australia	Breast	1505 women sent GP invitation to mammography. No control group.	68.8% of eligible women attended.	None reported.
Eilbert et al.^75^ Multiple interventions in Tower Hamlets, particularly including language- and culture-sensitive GP endorsement and reminders from primary care.	London, UK	Breast	No control group. Interventions carried out over a two-year period in a large urban area. Pre-intervention uptake compared to post-intervention.	Between 2005 and 2008, the period of the intervention, breast screening uptake increased from 45% to 63%.	Study took place in Tower Hamlets, inner north-east London, an area of high deprivation and ethnic diversity.
De Nooijer et al.^76^ GP vs. local health authority invitation.	Netherlands	Cervical	88,194 women invited by GP, 149,525 by local health authority. Non-randomized.	Significant 7.9% higher attendance with GP invitation.	Effect greatest among ethnic minorities, urban areas, and younger women.
Hermens et al.^77^ GP vs. local health authority invitation. Reminder vs. no reminder for non-responders.	Netherlands	Cervical	Non-randomized study, 9531 women invited by primary care or local health authority. Non-attenders sent reminders in some areas.	In women aged ≤ 45, 68% attendance for primary care invitations vs. 53% for local health authority invitations. In women aged > 45, 58% vs. 47%. Reminders increased participation by 7%–11%, depending on age and invitation source.	None reported.

GP: general practitioner; FOBT: faecal occult blood test; FIT: faecal immunochemical test; FIT: faecal immunochemical test.

### Interventions targeted specifically on non-participants

Results are shown in Table 4. Fifteen studies were identified, three in colorectal, eight in breast, and four in cervical cancer screening.^16,27–40^ Twelve of the fifteen studies found an increase in participation with the intervention; 3/6 telephone reminder studies had positive results, compared to 7/7 postal interventions (including two where the letter was from the subject's own GP), 1/1 study of combined telephone and postal reminders, and 1/1 which found increased participation with sending a second faecal occult blood test (FOBT) kit instead of a reminder letter. Typical effect sizes were of the order of a 10% increase in participation with reminder letters, and rather smaller effects with telephone reminders. Very large effects were noted for reminder letters compared with no further contact,^32^ but as the former is standard practice in the UK, there is no policy change implied by this result. Substantially greater participation was observed for second timed appointment for breast screening non-attenders, compared with open invitation to call and make an appointment.^30^ One study of an enhanced reminder letter, one of additional reminder letters, and two studies of telephone reminders reported positive results in underserved populations.^16,28,31,34^

**Table 4.** Interventions in non-participants.

Publication and intervention	Region	Cancer	Design	Results	Inequality dimension
Wardle et al.^16^ Reminder letter to non-participants enhanced to give more personal message.	England	Colorectal	Cluster randomized trial. 90,413 invitees randomized to receive usual reminder letter, 78,067 to receive enhanced reminder letter.	Significant improvement in uptake with intervention. Significant heterogeneity of effect by deprivation, with greater effect of intervention in more deprived groups. Increase in most deprived group was from 13% to 14%.	Formally designed to investigate significant difference in effect among deprivation categories.
Tinmouth et al.^39^ FOBT kit sent with reminder letter to non-participants.	Toronto, Canada	Colorectal	Cluster randomized trial: 2008 women randomized to receive kit (intervention), 1586 letter only (control).	Uptake 20% in intervention group vs. 10% in control.	Controlled for area-based measure of socioeconomic status.
Steele et al.^40^ Repeated invitations to non-participants.	Scotland, UK	Colorectal	Analysis of prevalence and incidence screening of adults aged 50–69. Three rounds of biennial colorectal screening using the guaiac faecal occult blood test.	Repeat invitations to those who do not take up the offer of screening increased the number of participants, from 54% to 86% at incidence screening.	None reported.
Atri et al.^28^ Telephone or, failing that, postal contact with non-attenders by GP reception staff.	London, UK	Breast	Controlled trial, randomized by general practice. 2064 women aged 50–64 who had failed to attend for breast screening.	Attendance in the intervention group was significantly better than in the control group (9% vs. 4%).	Study took place in Newham, inner north-east London, an area with significant deprivation and high ethnic diversity. Improvement was greatest in Indian women (19% vs. 5%).
Kearins et al.^29^ Telephone or home visits to non-attenders.	Birmingham, UK	Breast	Uncontrolled study of 548 persistent non-attenders identified in routine screening lists. Phone contact was attempted or a home visit was made. If the case was not resolved, a second appointment was made and further phone calls and home visits were attempted.	Phone calls and home visits resulted in only a moderate increase in breast cancer screening uptake. The initiative made it easier for women to request to be permanently withdrawn from the NHSBSP.	Urban setting. Otherwise, none reported.
Stead et al.^30^ Second timed appointments in non-attenders of initial breast screening second appointment.	UK	Breast	2229 women who had failed to attend and had not declined their first invitation to screening were randomized to receive an ‘open’ invitation asking them to telephone the screening unit for another appointment or to be given a second fixed appointment time.	Fixed appointment letter had a significantly greater uptake than the open invitation (23% vs. 12%).	Effect did not vary by socioeconomic status.
Turner et al.^31^ Letter signed by own GP with second invitation.	Aberdeen, UK	Breast	234 non-responders randomized to GP letter with second invitation, 231 to usual second invitation.	Significantly higher response with GP letter (21% vs. 10%).	None reported.
Fleming et al.^32^ Reminder letters.	Dublin, Ireland	Breast	Uncontrolled study of reminders for non-attenders.	Reminders increased uptake by 30%.	None reported.
Hayes et al.^33^ Second and third reminder letters.	Dublin, Ireland	Breast	Non-responders to an invitation for screening were re-invited by computer-generated letter to attend for screening six weeks after issue of the first invitation and a final invitation was issued at 12 weeks.	Issue of second mailed invitations to women in the target age for breast screening increased uptake from 61% to 79%. Third invitations were not cost-effective. Women aged 55–64 were more likely to respond to first, second, or third invitations than those aged less than 55.	No difference in effect by whether or not the invitee had private health insurance.
Hegensheid et al.^34^ Telephone reminders.	Germany	Breast	Non-attenders randomized to telephone counselling (2472) vs. written reminder (3005).	Significantly higher participation with telephone counselling (30% vs. 26%).	Effect present in different educational status groups.
Goelen et al.^27^ Telephone reminders.	Belgium	Breast	3880 women who had not attended for screening randomized to written invitation or written invitation plus telephone call.	22% attendance with telephone call vs. 18% without.	None reported.
Jensen et al.^35^ Additional GP-signed letter to non-attenders.	Aarhus, Denmark	Cervical	Cluster randomized trial. General practices were unit of randomization. 7527 non-attenders in intervention group, 7452 in control.	Significant (1%–2%) improvement in coverage in intervention group after nine months.	None reported.
Oscarsson et al.^36^ Personal contact, including telephone contact, of non-attenders.	Kalmar County, Sweden	Cervical	400 randomized to intervention, 400 to control.	118 (30%) women in study group had a smear, 74 (19%) in control (*p* < 0.001).	None reported.
Stein et al.^37^ Telephone call from nurse, celebrity endorsement, or letter from public health physician responsible for commissioning cervical screening to non-attenders.	Devon, UK	Cervical	Randomized trial: 285 women in each intervention group + 285 women in control group.	No significant effect of any intervention.	None reported.
Heranney et al.^38^ Letter vs. telephone call one year after non-response.	Alsace, France	Cervical	10,662 women randomized to a reminder letter or a telephone call.	No significant difference in response: 6.3% for telephone call, 5.8% for letter.	None reported.

GP: general practitioner; FOBT: faecal occult blood test.

### Varying invitation materials or strategy

Results are shown in Table 5. Eighteen intervention studies reported in 17 papers were identified, 11 in colorectal, four in breast, one in cervical, one in breast and cervical, one in melanoma screening.^10,16,17,41–45,47–52,56–58^ The 17 papers reported on evaluation of 20 interventions; some studies evaluated more than 1 intervention simultaneously. Thirteen interventions were observed to be associated with increased participation. Enclosing survey questionnaires with the invitation had little effect on participation, and one study found a significantly reduced participation with the intervention, with the reduction greatest in deprived areas.^42,56^ Fixed screening appointment times compared with open invitations increased participation by around 20% (in absolute terms), and one study found a 3% increase when the invitation included the opportunity to switch to an evening or weekend appointment time for breast cancer screening.^10,51^ The latter took place in Manchester and Bristol, both of which included areas of deprivation.^10^ Out of five studies (in four publications) of varying the information with the invitation, only one found an increase in participation with the intervention.^16,17,43,52^ Two studies of advance notice of the screening invitation found an increase in participation.^41,43^ One study found an increased participation rate with the offer of a health check,^48^ and another found no increase with the offer of counselling.^50^ Two out of three evaluations of mass media campaigns found an increase in participation.^17,44,45^ Two studies found that direct mailing of FOBT kits led to higher participation than the request to collect a kit at primary care.^57,58^ One found increased participation with the mailing of a pack of equipment with the FOBT kit, including latex gloves and stool catchers to facilitate collection of stool samples.^17^

**Table 5.** Enhanced invitation materials/varying invitation strategy.

Publication and intervention	Region	Cancer	Design	Results	Inequality dimension
Watson et al.^56^ Enclosure of research questionnaires with screening invitation.	Midlands and north-west of England	Colorectal	People invited for screening randomized to receive or not to receive an additional research study questionnaire, consent form and study information.	Receiving study documents significantly reduced screening uptake.	Reduction was greatest in deprived areas.
Wardle et al.^16^ Simplified information leaflet.	England	Colorectal	Cluster randomized trial. 79,104 invitees randomized to usual information, 84,421 usual information plus simplified leaflet.	No significant effect on uptake. No significant difference in effect among deprivation categories.	Formally designed to investigate significant difference in effect among deprivation categories.
Wardle et al.^16^ Narrative leaflet with past screenees' stories and quotes.	England	Colorectal	Cluster randomized trial. 76,695 invitees randomized to usual information, 73,722 to usual information plus narrative leaflet.	No significant effect on uptake. No significant difference in effect among deprivation categories.	Formally designed to investigate significant difference in effect among deprivation categories.
Wardle et al.^47^ Additional health education brochure with screening invitation.	London, UK	Colorectal	Adults ages 55–64 in a ‘harder-to-reach’ group randomized either to receive an intervention brochure or to a standard invitation group.	Compared with controls, the intervention group had a higher level of attendance (54% vs. 50%).	Intervention more effective in lower socioeconomic tertiles.
Mant et al.^48^ Addition of general health check to FOBT.	Oxford, UK	Colorectal	404 subjects randomized to FOBT only, 1084 to some combination of FOBT with health check.	Highest participation observed when FOBT kit was sent with an invitation to health check (44% vs. 43%).	None reported.
Senore et al.^41^ Advance notification letter.	Italy	Colorectal	44,198 invitees randomized to standard invitation; advance notification letter; advance notification letter with offer of GP consultation.	Advance notification significantly increased participation (38% vs. 34%). Simple letter achieved same result as letter with offer of GP consultation.	None reported.
Helander et al.^42^ Health, lifestyle survey one year prior to screening.	Finland	Colorectal	15,748 invited to colorectal screening, of whom 5185 randomized to questionnaire survey.	In survey group 57% participated; in non-survey group, 60%.	None reported.
Giorgi Rossi et al.^57^ Mailing of FOBT kit compared to invitation to collect at primary care.	Central Italy	Colorectal	Two randomized trials: one in 3196 previous responders, one in 4219 non-responders to first invitation.	Significantly higher response with direct mailing of kit (63% vs. 57% in previous responders; 15% vs. 11% in non-responders).	None reported.
Van Roosbroeck et al.^58^ Mailed vs. GP provided FOBT kits.	Flanders, Belgium	Colorectal	Not clear if randomized. 11,490 subjects mailed kit. 8052 sent letter asking them to collect kit from GP.	52% of mailed kit group participated, 28% of GP group.	Difference was observed in subgroups by urban/rural status.
Cole et al.^43^ Advance notification letter, risk information, advocacy from previous participants.	South Australia	Colorectal	Randomized trial in subjects eligible for FIT testing: 600 usual invitation; 600 additional risk information; 600 advocacy from previous screenees; 600 advance notification letter.	Significant increase in participation only in advance notification group (40%, 40%, 36%, and 48%, respectively).	None reported.
White et al.^17^ Additional flyer with FOBT kit, including endorsement by a major cancer charity, combined with enhanced FOBT pack and/or advertising campaign.	London, UK	Colorectal	Non-randomized comparative study. 200,939 invitees received endorsement flyer, compared with 177,386 contemporaneous invitees who received no intervention. Of those with flyer, 13,655 also received an enhanced FOBT pack with rubber gloves and other equipment. 9830 additionally were targeted by an advertising campaign.	Participation rates were: no intervention 44%; flyer alone 43%; flyer plus pack 45%; flyer plus advertising 46%; all three interventions 50%. Latter three significant.	Study took place in a region of high mobility and low participation.
Offman et al.^10^ Out of office hours screening appointments.	UK	Breast	Four-armed randomized trial of women invited for routine breast screening randomized (3:1:1:1) to one of these screening invitations: standard office hour appointment, office hour appointment with the option to change to an out-of-hours appointment, weekday evening appointment, or weekend appointment.	The optimum strategy for improving attendance at breast screening was to offer a traditional office hour appointment and including in the letter of invitation an option to change to an evening or weekend appointment if wished (76% vs. 73%).	Trial took place in Manchester and Bristol, each centre containing urban areas of significant deprivation.
Banks et al.^49^ Enclosure of a questionnaire with invitation to breast screening.	England	Breast	Randomized study of 6400 women invited for routine screening mammography were individually randomized to receive either the usual breast screening invitation alone, or to receive the usual invitation accompanied by a self-administered questionnaire.	Screening uptake was not affected by the intervention.	Lower uptake in older women. Otherwise, none reported.
Giordano et al.^50^ Multiple interventions including enhanced invitation.	Italy	Breast	5360 women randomized to: (1) usual invitation (2) enhanced written information (3) offer of counselling (4) invitation to contact the centre for information and arrangement of appointment.	Participation rates were: (1) 36.5% (2) 39.9% (3) 35.8% (4) 16.5%.	No significant variation of effect among socioeconomic groups.
Page et al.^44^ Mass media campaign in Italian language.	New South Wales, Australia	Breast	Italian language newspaper and radio promotion.	No change in uptake rates between pre- and post-intervention.	Italian-speaking women in Australia, considered a hard-to-reach group.
Nguyen et al.^45^ Multiple outreach intervention.	California and Texas, USA	Cervical	1004 Vietnamese women in one area targeted with multiple outreach activities. 1005 women in control area received usual care. Non-randomized.	Significantly greater pap test coverage in intervention area (84% vs. 71%).	Study took place in a developing country.
Segnan et al.^51^ Fixed vs. open appointments, GP vs. programme invitation.	Italy	Breast and cervical	16,454 women randomized to: (A) GP invitation, fixed appointment (B) GP invitation, open-ended (C) programme invitation, fixed appointment (D) extended GP letter, fixed appointment.	Highest response in (A) and (D). No difference between (A) and (D). Groups (B) and (C) had significantly lower attendance. (A) 42% (B) 21% (C) 36% (D) 43%	None reported.
Youl et al.^52^ Addition of explanatory brochure to invitation letter.	Australia	Skin	661 randomized to usual letter, 661 to letter plus brochure.	No significant effect on participation.	None reported.

GP: general practitioner; FOBT: faecal occult blood test; FIT: faecal immunochemical test.

### Direct contact interventions

Results are shown in Table 6. Five studies were identified, one in colorectal, three in breast, and one in cervical cancer screening.^46,53–55,59^ Three studies were of home visits, two finding increased participation associated with the intervention.^53,55,59^ One study of direct telephone contact by a health professional found an increase in participation with the intervention.^54^ One study of opportunistic promotion of breast screening at clinic attendances for other reasons found an increase in participation, especially in women of low socioeconomic status.^46^

**Table 6.** Direct contact interventions.

Publication and intervention	Region	Cancer	Design	Results	Inequality dimension
Courtier et al.^59^ Home visit.	Barcelona, Spain	Colorectal	1060 randomized to mailed kit and letter, 965 to home visit with kit.	Significantly higher participation in home visit group (58% vs. 36%).	Urban population.
Hoare et al.^53^ Home visit.	Manchester, UK	Breast	The control group received no visits. The study population comprised all women with Asian names, from a batch of general practices where high proportions of patients were Asian, who were invited for screening.	No difference in attendance was found between the intervention and control groups.	This study took place in an Asian population in an urban environment including areas of substantial deprivation.
Segura et al.^54^ Direct contact by health professional.	Barcelona, Spain	Breast	564 women randomized to programme invitation letter, primary care invitation letter, or direct contact.	Highest participation in direct contact group (52%, 56%, 64% in the three groups, respectively).	Increased participation was strongest in women of lower educational status.
Taylor et al.^46^ Promotion at routine clinic visit for other medical reasons.	Seattle, USA	Breast	232 women randomized to promotion, 82 to usual care.	49% in intervention group participated, 22% in control.	Inner city population. Significant effect in black women and in those with and without health insurance.
Chalapati et al.^68^ Home visit.	Thailand	Cervical	Geographic zone randomization. 158 women in intervention group, 146 in control.	Non-significant increase in coverage in intervention zone (43% vs. 35%).	Took place in a developing country with corresponding levels of education and socioeconomic status.

### Varying the screening test

Results are shown in Table 7. Ten studies were identified, three in colorectal^60–62^ and seven in cervical screening.^63–69^ Faecal immunochemical testing (FIT) yielded 15%–20% higher participation rates than either colonoscopy^61^ or guaiac FOBT.^62^ The improvements over both colonoscopy and guaiac FOBT did not vary substantially by age, sex, or ethnicity. One study in Germany comparing conventional with capsule colonoscopy found a small increase in participation with the latter.^60^ All the cervical screening studies were of the offer of human papillomavirus (HPV) self-sampling, usually to women with a history of non-participation,^63–69^ and all found increased participation with the offer of self-sampling, typically of the order of 10%. The one study reporting effects by socioeconomic status found the intervention equally effective in different socioeconomic groups.^66^ The intervention is effective in previous non-participants, who are frequently characterized by lower socioeconomic status or specific ethnic profiles.^5^

**Table 7.** Alternative screening tests.

Publication and intervention	Region	Cancer	Design	Results	Inequality dimension
Gupta et al.^61^ Invitation to FIT/colonoscopy vs. opportunistic screening.	Texas, USA	Colorectal	Subjects randomized: 1593 to invitation to FIT; 479 to invitation to colonoscopy; 3898 to opportunistic offer of screening at primary care consultation. Effect estimated in each ethnic group.	Participation significantly higher for FIT (40.7%) than for colonoscopy (24.6%) or opportunistic screening (12.1%).	Effect observed in all ethnic groups.
Groth et al.^60^ Capsule colonoscopy.	Lower Saxony, Germany	Colorectal	2150 persons offered choice of conventional or capsule colonoscopy.	90 (4.2%) underwent capsule colonoscopy and 34 (1.6%) conventional.	Effect stronger in men than in women. Otherwise, none reported.
Santare et al.^62^ FIT test vs. gFOBT.	Latvia	Colorectal	3 × 2 factorial trial, 15,000 subjects randomized. 5000 gFOBT; 5000 FIT FOB Gold; 5000 FIT OC-Sensor. 7500 randomized to receive an advance notification letter.	Uptake was 31% for gFOBT, 45% for FIT FOB Gold, 47% for FIT OC-Sensor. Uptake was 39% without advance letter, 42% with advance letter.	Effects observed in subgroups of age, sex and urban/rural status.
Szarewski et al.^66^ HPV self-sampling.	London	Cervical	3000 women randomly selected from persistent non-responders were randomized 1:1 to either receive an HPV self-sampling kit or a further invitation to attend for cervical cytology.	Significantly more women responded to self-sampling than the control (10% vs. 5%).	Effect did not vary by socioeconomic status.
Haguenoer et al.^63^ HPV self-sampling.	Indre-et-Loire, France	Cervical	Previous non-attenders for Pap smear screening randomized to: (1) no intervention (2) further invitation for Pap smear (3) sent self-sampling kit.	Response rates: (1) 9.9% (2) 11.7% (3) 22.5%.	Similar effects in age subgroups. Otherwise, none reported.
Broberg et al.^64^ HPV self-sampling.	Western Sweden	Cervical	Non-attenders for two rounds of screening randomized to: 800 to HPV self-test; 4000 to telephone call; 4000 to standard invitation.	Response in self-tests arm 24.5%, significantly higher than other two arms (control 18%).	Effect stronger in older women.
Darlin et al.^67^ HPV self-sampling.	Sweden	Cervical	Women unscreened for nine years randomized: 1000 to HPV self-sampling; 500 to flexible outpatient appointments.	Significantly higher participation for self-sampling (14.7%) compared to flexible OP appointments (4.2%).	None reported.
Virtanen et al.^68^ HPV self-sampling.	Finland	Cervical	Screening non-attenders randomized to self-sampling (2397) or additional invitation (6302).	Significantly higher response to self-sampling (76% vs. 65%).	Effect similar in different language groups.
Gök et al.^69^ HPV self-sampling.	Netherlands	Cervical	27,792 previous non-attenders randomized to HPV self-sampling, 281 to routine recall.	Significantly higher response to self-sampling (26.6% vs. 16.4%).	None reported.
Giorgi Rossi et al.^65^ HPV self-sampling.	Italy	Cervical	14,041 non-attenders for cervical screening randomized to: (1) usual reminder letter (2) self-sampling kit sent to home (3) opportunity to pick up self-sampling kit at pharmacy.	Participation: (1) 12% (2) 22% (3) 12%.	Significant difference in effect among centres.

FOBT: faecal occult blood test; HPV: human papillomavirus; OP: out patient; gFOBT: guaiac-based faecal occult blood test; FIT: faecal immunochemical test.

## Discussion

A number of results seem to be observed consistently between studies and across different countries and health systems. Both pre-screening reminders and GP endorsement led to higher participation rates (albeit modest increases) and were observed to do so in deprived and otherwise underserved populations.^16–18,21,22,74,75,79^ More personalized reminders for non-participants, whether by enhanced written materials or telephone contact (notably from primary care), were effective in increasing participation. These interventions too were successful in populations of low socioeconomic status.^16,30,33,40^ Primary care endorsement and enhanced reminders for non-participants would incur almost no expense (other than the cost of screening larger numbers of people). In the UK, they might be expected to result in small, but arguably worthwhile, increases in participation. Larger increases might be expected in more deprived populations with lower current participation rates.^16^

The choice of screening test itself is associated with participation. FIT is clearly more popular than other bowel screening modalities and was observed to increase participation by 15%–20%. HPV self-sampling raised participation rates, notably in previous non-participants, by around 10%. This is likely to be effective in socioeconomic or ethnic populations traditionally less easy to reach with cervical screening.^62^ Less consistent results were observed for different invitation strategies and home visits. Inclusion of questionnaires for research along with the invitations seemed to have a negative effect.^38^

It is accepted that interventions to promote cancer screening should be non-coercive and should respect the principle of informed choice.^80^ However, it is also the case that there is a strong socioeconomic gradient in participation in screening, with lower participation being associated with lower socioeconomic status,^3^ and there is evidence that non-participants often report not having read the information provided.^81^ The search for interventions to remedy this would, therefore, seem to be ethically justified.

The classification of studies by intervention was arbitrary. For example, most of the HPV self-sampling studies included in Table 7 as interventions varying the screening test could have been included in Table 4 as interventions targeting non-participants. Similarly, some of the latter could have been included as primary care endorsement studies. However, the results are generally clear. It is also worth noting that, in classifying the studies, a degree of oversimplification was inevitable, in that some multi-component interventions have been classified into one category or another. One example is the study by Bell et al.,^72^ which is classified as primary care endorsement, but which also included multilingual approaches and offers of transport to screening centre.

The magnitude of effects varied considerably, even within intervention types. The effects tended to be larger in environments where participation rates were relatively low. For example, reminder studies showing particularly large effects on participation were that of Baker et al.^21^ in Chicago, where opportunistic rather than organized screening was taking place, and one in inner north-east London, where there are large deprived populations, high levels of ethnic diversity, and usually low screening participation rates.^8,22^ Similarly, in the primary care endorsement studies, greatest effects were seen in populations with previously low participation.^72,75^

In the studies identified, the patient navigation approach,^82^ whereby a ‘navigator’ guides the patient/invitee through the complexities of a screening, diagnostic, or therapeutic process, was largely absent. However, some of the telephone interventions involved detailed scripts and briefing of the staff, so that they were able to answer questions; indeed, one such study in the context of bowel cancer screening showed good results.^22^ The concept of patient navigation is already established in the USA and may well spread to Europe in the immediate future.

We did not sub-classify the studies by design or quality but have noted in the tables whether the studies were randomized trials or observational studies. Of the 71 intervention studies, 52 (73%) were randomized, either by individual or cluster. The majority of positive results were seen in randomized studies.

As noted in the Methods section, due to time and resource considerations, we restricted our search to peer-reviewed publications listed in PubMed and adopted an unconventional expanding search strategy rather than a comprehensive search followed by successive narrowing by abstract and paper review. Although we only ceased the search expansion when it yielded relatively small numbers of potentially eligible publications and built in a safeguard by specifying that the search had to include a number of key publications, it is possible that some eligible material has been missed.^83^ Watt et al.^84^ advocate not a standard methodology for rapid review, but clear reporting and transparency with respect to the methods used. We have tried to adhere to this in terms of the description in the Methods section and the information in Table 1. It would be interesting if another group with the time and resources carried out a traditional systematic review, to see what, if anything, we have missed.

## Conclusion

Interventions which were found most consistently to improve participation in cancer screening, including in underserved populations, were pre-screening reminders, general practice endorsement, more personalized reminders for non-participants, and offering a more acceptable screening test in cervical and bowel screening, both of which may suffer from social and cultural taboos.
